# Supplementary material for: The microeconomic impact of out-of-pocket medical expenditure on the households of cardiovascular disease patients in general and specialized heart hospitals in Ibadan, Nigeria
Source: PLoS One. 2022 Jul 18;17(7):e0271568. doi: 10.1371/journal.pone.0271568 (PMC9292125; doi:10.1371/journal.pone.0271568)
Supplement: Attachment — Submitted filename: tao Comments.docx [file pone.0271568.s002.docx]

**The microeconomic impact of out-of-pocket medical expenditure on the households of cardiovascular disease patients in general and specialized heart hospitals in Ibadan, Nigeria**

**Comments**

1. Change Vancouver formatting style from (9) to [9]. [ ] is the accepted formatting style for PlosOne.
2. On line 89. Consider changing, “*This oftentimes undermine the goal*….” To: “*These oftentimes undermine the goal*….” Or “*This oftentimes undermines the goal*….”
3. On page 499 In Authors’ contributions: Keep authors initials consistent with author names i.e Please remove FA, OL and KO and replace with KIFA. AOL and KOO.
4. In Acknowledgement section: It is always desirable to acknowledge the reviewers also for their roles in improving the paper
5. In the Tables, (clearly specify by adding “Ref” category beside all reference categories e.g <45 (Ref); Male (Ref) etc.
6. In Table 1, Under Household Positions, include what categories of people you classify as “*others*’ under the table as a foot note. And please change it from “*Other*” to “*Others*”.
7. Ensure that tense throughout the results section is reported in past tense. The tense used for reporting in the results seems to alternate between present and past tenses. For instance, on line 264…. “*Relating to the occupational distribution of the respondents, 477, representing 64.11%* ***are*** *gainfully employed*” should be rephrased as, “*Relating to the occupational distribution of the respondents, 477, representing 64.11%* ***were*** *gainfully employed*”
8. On Line 141, the assertion that Ibadan is renowned to be the biggest city in West Africa only holds true for land mass, not for population or other parameters. Readers may be confused if this is not clearly stated. Please add, “with respect to land mass” to Line 141
9. On Line 184…direct is repeated twice….” *only the OOP expenditures (both direct and direct costs) pertaining*” …. ought to be,” *…only the OOP expenditures (both direct and indirect costs) pertaining…*
10. On Line 110, “*But, there has been no disease-specific studies examining the household-level microeconomic impact of OOP payments for the treatment of CVDs*” is a pretty strong assertion to make. I believe what authors mean to say is that this kind of study has not been evaluated in detail yet in SSAs or in developing countries such as Nigeria before. I suggest revising that statement to read, “*But, there has been no disease-specific studies examining the household-level microeconomic impact of OOP payments for the treatment of CVDs* ***in Nigeria***”.
