# Supplementary material for: The microeconomic impact of out-of-pocket medical expenditure on the households of cardiovascular disease patients in general and specialized heart hospitals in Ibadan, Nigeria
Source: PLoS One. 2022 Jul 18;17(7):e0271568. doi: 10.1371/journal.pone.0271568 (PMC9292125; doi:10.1371/journal.pone.0271568)
Supplement: Attachment — Submitted filename: Response to Reviewers.docx [file pone.0271568.s003.docx]

May 21, 2022

Plos One

Dear Editor,

**Subject**: PONE-D-21-32628

The microeconomic impact of out-of-pocket medical expenditure on the households of cardiovascular disease patients in general and specialized heart hospitals in Ibadan, Nigeria

Thank you for your letter/email and the opportunity to revise our manuscript.

We have carefully considered the very valuable suggestions and comments offered by the editor and reviewers. We can say that those comments have been immensely helpful in improving the quality of our manuscript.

We hereby include the editorial comments as well as the reviewer comments below and provided a point-by-point response to all the comments. We included the section, lines and pages where the edits/revisions have been made.

The manuscript has also been scrutinized for the requirements in the author instructions, as well. All the authors contributed and agreed to the revisions.

Thank you very much

Regards,

Dr. Folashayo Adeniji

On behalf of the authors

**Editor Comments:**

**Journal requirements:**

When submitting your revision, we need you to address these additional requirements.

1. Please ensure that your manuscript meets PLOS ONE's style requirements, including those for file naming. The PLOS ONE style templates can be found at

https://journals.plos.org/plosone/s/file?id=wjVg/PLOSOne_formatting_sample_main_body.pdf and

<https://journals.plos.org/plosone/s/file?id=ba62/PLOSOne_formatting_sample_title_authors_affiliations.pdf>.

Response: The authors read the Journal’s guidelines for manuscript preparation and our manuscript was formatted accordingly.

2. We note that the grant information you provided in the ‘Funding Information’ and ‘Financial Disclosure’ sections do not match. When you resubmit, please ensure that you provide the correct grant numbers for the awards you received for your study in the ‘Funding Information’ section.

Response: This is noted and will be corrected when re-submitting

3. Thank you for stating the following in the Acknowledgments Section of your manuscript:

“This research was supported by the Consortium for Advanced Research Training in Africa (CARTA). CARTA is jointly led by the African Population and Health Research Center and the University of the Witwatersrand and funded by the Carnegie Corporation of New York (Grant No--B 8606.R02), Sida (Grant No:54100113), the DELTAS Africa Initiative (Grant No: 107768/Z/15/Z) and Deutscher Akademischer Austauschdienst (DAAD). The DELTAS Africa Initiative is an independent funding scheme of the African Academy of Sciences (AAS)’s Alliance for Accelerating Excellence in Science in Africa (AESA) and supported by the New Partnership for Africa’s Development Planning and Coordinating Agency (NEPAD Agency) with funding from the Wellcome Trust (UK) and the UK government. The statements made and views expressed are solely the responsibility of the Fellow”

We note that you have provided funding information that is not currently declared in your Funding Statement. However, funding information should not appear in the Acknowledgments section or other areas of your manuscript. We will only publish funding information present in the Funding Statement section of the online submission form.

Please remove any funding-related text from the manuscript and let us know how you would like to update your Funding Statement. Currently, your Funding Statement reads as follows:

“"This research was supported by the Consortium for Advanced Research Training in Africa (CARTA). CARTA is jointly led by the African Population and Health Research Center and the University of the Witwatersrand and funded by the Carnegie Corporation of New York (Grant No--B 8606.R02), Sida (Grant No:54100113), the DELTAS Africa Initiative (Grant No: 107768/Z/15/Z) and Deutscher Akademischer Austauschdienst (DAAD). The DELTAS Africa Initiative is an independent funding scheme of the African Academy of Sciences (AAS)’s Alliance for Accelerating Excellence in Science in Africa (AESA) and supported by the New Partnership for Africa’s Development Planning and Coordinating Agency (NEPAD Agency) with funding from the Wellcome Trust (UK) and the UK government. The statements made and views expressed are solely the responsibility of the Fellow".

NO - Include this sentence at the end of your statement: The funders had no role in study design, data collection and analysis, decision to publish, or preparation of the manuscript.”

Please include your amended statements within your cover letter; we will change the online submission form on your behalf.

Response: This is noted. The funding statement has been removed from the manuscript and included in the cover letter.

4. We note that you have indicated that data from this study are available upon request. PLOS only allows data to be available upon request if there are legal or ethical restrictions on sharing data publicly. For more information on unacceptable data access restrictions, please see http://journals.plos.org/plosone/s/data-availability#loc-unacceptable-data-access-restrictions.

In your revised cover letter, please address the following prompts:

a) If there are ethical or legal restrictions on sharing a de-identified data set, please explain them in detail (e.g., data contain potentially sensitive information, data are owned by a third-party organization, etc.) and who has imposed them (e.g., an ethics committee). Please also provide contact information for a data access committee, ethics committee, or other institutional body to which data requests may be sent.

b) If there are no restrictions, please upload the minimal anonymized data set necessary to replicate your study findings as either Supporting Information files or to a stable, public repository and provide us with the relevant URLs, DOIs, or accession numbers. For a list of acceptable repositories, please see <http://journals.plos.org/plosone/s/data-availability#loc-recommended-repositories>.

Response: The data used for this study has been uploaded.

We will update your Data Availability statement on your behalf to reflect the information you provide.

5. PLOS requires an ORCID iD for the corresponding author in Editorial Manager on papers submitted after December 6th, 2016. Please ensure that you have an ORCID iD and that it is validated in Editorial Manager. To do this, go to ‘Update my Information’ (in the upper left-hand corner of the main menu), and click on the Fetch/Validate link next to the ORCID field. This will take you to the ORCID site and allow you to create a new iD or authenticate a pre-existing iD in Editorial Manager. Please see the following video for instructions on linking an ORCID iD to your Editorial Manager account: <https://www.youtube.com/watch?v=_xcclfuvtxQ>.

Response: The ORCD ID of the corresponding author has been added to the Editorial Manager

6. Your ethics statement should only appear in the Methods section of your manuscript. If your ethics statement is written in any section besides the Methods, please delete it from any other section.

Response: The ethics statement has been removed from the acknowledgement and taken to the methods section.

Please review your reference list to ensure that it is complete and correct. If you have cited papers that have been retracted, please include the rationale for doing so in the manuscript text, or remove these references and replace them with relevant current references. Any changes to the reference list should be mentioned in the rebuttal letter that accompanies your revised manuscript. If you need to cite a retracted article, indicate the article’s retracted status in the References list and also include a citation and full reference for the retraction notice.

Response: We have looked at the references again and made necessary revisions. Kindly refer to the reference section of the manuscript.

Reviewer #1 Comments

1. Change Vancouver formatting style from (9) to [9]. [ ] is the accepted formatting style for PlosOne.

Response: Square brackets have been used in line with journal requirements.

2. On line 89. Consider changing, “*This oftentimes undermine the goal*….” To: “*These oftentimes undermine the goal*….” Or “*This oftentimes undermines the goal*….”

Response: This has been revised to reflect the suggestion of the reviewer. See line 90.

3. On page 499 In Authors’ contributions: Keep authors initials consistent with author names i.e Please remove FA, OL and KO and replace with KIFA. AOL and KOO.

Response: This has been revised. See line 508

4. In Acknowledgement section: It is always desirable to acknowledge the reviewers also for their roles in improving the paper

Response: The reviewer has been acknowledged in our acknowledgement section.

5. In the Tables, (clearly specify by adding “Ref” category beside all reference categories e.g <45 (Ref); Male (Ref) etc.

Response: The reference category has been identified. See Tables 5a-6b.

6. In Table 1, Under Household Positions, include what categories of people you classify as “*others*’ under the table as a foot note. And please change it from “*Other*” to “*Others*”.

Response: This has been included. See Table 1

7. Ensure that tense throughout the results section is reported in past tense. The tense used for reporting in the results seems to alternate between present and past tenses. For instance, on line 264…. “*Relating to the occupational distribution of the respondents, 477, representing 64.11%* ***are*** *gainfully employed*” should be rephrased as, “*Relating to the occupational distribution of the respondents, 477, representing 64.11%* ***were*** *gainfully employed*”

Response: Result section has been revised accordingly to ensure that all findings are consistently reported in past tense. See lines 265-362

8. On Line 141, the assertion that Ibadan is renowned to be the biggest city in West Africa only holds true for land mass, not for population or other parameters. Readers may be confused if this is not clearly stated. Please add, “with respect to land mass” to Line 141

Response: This statement has been revised to reflect the suggestion of the reviewer.

9. On Line 184…direct is repeated twice….” *only the OOP expenditures (both direct and direct costs) pertaining*” …. ought to be,” *…only the OOP expenditures (both direct and indirect costs) pertaining…*

Response: This has been corrected accordingly

10. On Line 110, “*But, there has been no disease-specific studies examining the household-level microeconomic impact of OOP payments for the treatment of CVDs*” is a pretty strong assertion to make. I believe what authors mean to say is that this kind of study has not been evaluated in detail yet in SSAs or in developing countries such as Nigeria before. I suggest revising that statement to read, “*But, there has been no disease-specific studies examining the household-level microeconomic impact of OOP payments for the treatment of CVDs* ***in Nigeria***”.

Response: As suggested by the reviewer, “in Nigeria” has been included to show that the assertion relates to Nigeria.

Overall, the manuscript was read by an expert editor and corrections were made were needed.

The author wish to thank the Editor and the reviewers for their valuable comments.
